# Tacrolimus-Induced Recurrent Acute Coronary Syndrome Due to an Unknown Mechanism

**DOI:** 10.7759/cureus.63266

**Published:** 2024-06-27

**Authors:** Sebastian L Manuel, Jenna Sapone, Frank Lin

**Affiliations:** 1 Internal Medicine, St. Luke's Hospital, Easton, USA

**Keywords:** acute coronary syndrome, chest pain, vasospasm, elevated troponin, tacrolimus

## Abstract

Tacrolimus, a potent immunosuppressive agent widely used in solid organ transplantation, has been associated with numerous harmful side effects. We report an interesting case of a patient who is status post liver transplantation and who has been maintained on tacrolimus for the past 10 years. She presented in the hospital with a ST-segment elevation myocardial infarction (STEMI), with normal coronary angiography. The patient was found to have a markedly elevated tacrolimus level. After decreasing the dose of tacrolimus and starting isosorbide mononitrate, the patient's symptoms resolved, and the patient had no recurrence of symptoms.

## Introduction

Tacrolimus is a calcineurin inhibitor that ultimately restricts interleukin 2 production. It has been a cornerstone of immunosuppressive therapy in solid organ transplantation due to its potent immunosuppressive properties [1]. Many immunosuppressive agents exhibit direct effects on multiple systems, including cardiovascular, renal, neurological, hematologic, and endocrine. In this context, we present a case of tacrolimus-induced coronary artery vasospasm in a liver transplant recipient and review the relevant literature to underscore the clinical implications and management strategies associated with this rare but clinically significant complication of tacrolimus therapy.

## Case presentation

**Figure 1:**
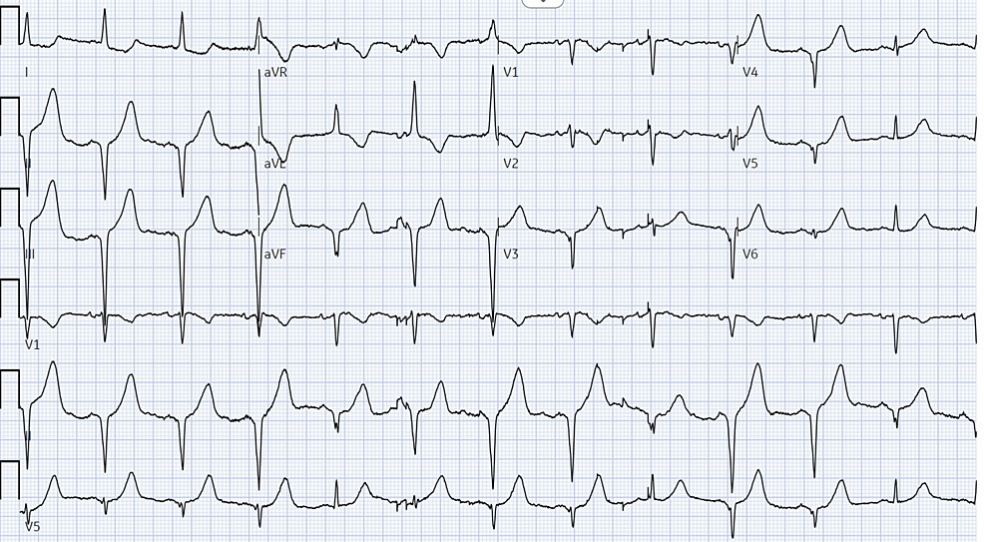
ST elevations of leads II, III, AVF

A 74-year-old female, with a past medical history of hepatocellular carcinoma secondary to hepatitis C status post-liver transplant on tacrolimus, hypertension on nifedipine and metoprolol, and anxiety on lorazepam, presented to the emergency department complaining of severe crushing substernal chest pain with radiation to the left arm with associated diaphoresis on exertion that was partially relieved with nitroglycerin. On arrival at the hospital, the patient was hemodynamically stable. An electrocardiogram (EKG) showed ST elevations on the inferior leads (Figure 1). High-sensitivity troponins peaked at 3,279 ng/L (normal <4 ng/L). Emergent cardiac catheterization demonstrated no coronary arterial disease. The patient was pain-free by the time she arrived at the catheterization laboratory. An echocardiogram demonstrated left ventricular ejection fraction normal at 65% with mild grade 1 diastolic dysfunction. She denied smoking, alcohol consumption, or drug use and had no prior cardiac history. The patient was asymptomatic with the resolution of elevated troponins; she was discharged home without any adjustments to her current medication regimen. At that time, there was no clear cause for the acute coronary syndrome event. 

One month later, she returned to the emergency department with recurrent, severe crushing substernal chest pain that was relieved by nitroglycerin. Troponins again were markedly elevated and peaked at 12,937 ng/L (normal <4 ng/L). EKG was unremarkable. Tacrolimus levels were noted to be supratherapeutic at 16.1 ng/mL. The patient was initially started on a heparin drip and ticagrelor; however, given recently normal cardiac catheterization and elevated tacrolimus levels, there was a suspicion of tacrolimus-induced vasospasm. Cardiology was consulted, and the heparin drip and ticagrelor were discontinued. The patient was started on isosorbide mononitrate in addition to her existing medications. It was discovered that her hepatologist had recently decreased the tacrolimus regimen due to gastrointestinal (GI) intolerance. About a month later, her GI symptoms resolved, and she was placed back on her original tacrolimus dosing. Throughout adjustments in tacrolimus, she was monitored closely and remained therapeutic. The following month is when the patient first presented due to chest pain. Before discharge, the dose was lowered to 0.5 mg twice a day. Tacrolimus blood level trended back down to 6.4 ng/mL before discharge. She has not had a recurrence of chest pain since those changes were made.

## Discussion

This patient was suspected to have a coronary artery vasospasm, in the setting of supratherapeutic tacrolimus levels. This can be supported by nitrate-responsive angina and transient ischemic EKG changes, which are criteria for diagnosing vasospastic angina [2]. A coronary angiogram was completed, which did not reveal atherosclerotic disease. Unfortunately, provocative maneuvers were not done for this case, which is usually recommended to help with diagnosis [2]. However, its resolution after tacrolimus dose adjustments and response to nitrates point toward a vasospastic picture.

Coronary artery vasospasm is implicated in the pathogenesis of ischemic heart disease and can be induced by many medications, most commonly cocaine. Other agents that have been shown to cause this phenomenon include catecholamines (epinephrine, norepinephrine, isoproterenol, dopamine, dobutamine), parasympathomimetic agents (acetylcholine, methacholine, pilocarpine), and rarely tacrolimus [3,4]. The incidence of coronary artery vasospasm is not readily available in the literature and is noted to be quite rare. It has, however, been documented with multiple case reports and has also been associated with cerebral artery vasospasms [5,6]. The pathophysiology of tacrolimus-induced coronary artery vasospasm is thought to be due to high concentrations of the drug binding to FK-binding protein (FKBP), which then forms a complex and inhibits calcineurin [7]. Calcineurin is a calcium-dependent phosphatase; thus, inhibition results in an increase in intracellular calcium concentration and resultant smooth muscle contraction. Calcineurin inhibitors such as tacrolimus and cyclosporine undergo extensive first-pass metabolism and clearance by the liver, thus leading to toxic levels [8].

Tacrolimus is the foundation of immunosuppressive medications for solid organ transplant recipients and most often the maintenance after liver transplantation. It has been superior to cyclosporine in terms of survival, graft loss, acute rejection, and steroid-resistant rejection within the first year [9]. It can be a difficult drug to manage due to its narrow therapeutic window in addition to the extensive metabolism undergone by the cytochrome P450 system. Pharmacokinetic drug interactions have been notorious for leading to toxicity. Many adverse reactions have been reported, which include neurotoxicity, renal dysfunction, hyperglycemia, infection, and malignancy [10]. Once a rarity, cardiotoxic effects have become more common and continue to be reported. Satisfactory tacrolimus trough levels after one year are typically from 3 to 5 ng/mL. Toxicity of tacrolimus commonly presents as acute renal failure; however, may also present with more nonspecific symptoms of headache or tremors. Unfortunately, there is no antidote to combat toxicity. Studies have shown drug-drug interactions with tacrolimus and nifedipine, as both are primarily metabolized by cytochrome P450 3A4 and 3A5 enzymes [11]. One drug interaction study reported any drugs known to be the substrates of the CYP3A inhibited the metabolism of tacrolimus, leading to increased blood concentrations. Among those agents were diltiazem, erythromycin, fluconazole, nifedipine, prednisolone, rifampin, and cyclosporine [12]. In the heart, immunosuppressive agents modulate cardiac hypertrophy, mitochondrial function, and arrhythmia risk, while in the vasculature, they influence vessel remodeling, circulating lipids, and blood pressure [12].

Common causes or triggers depicted in the literature remain multifactorial, but the strongest risk factor is cigarette smoking. Other triggers have also been identified, including cold exposure, psychological stressors, hyperventilation, alcohol consumption, stimulants, and marijuana abuse. Research has also demonstrated chemotherapy as a cause of coronary vasospasms, and studies suggest that those therapies induce vascular endothelial damage, leading to ischemia secondary to coronary artery vasospasm [13]. Infectious and inflammatory etiologies have also played a role in the induction of coronary vasospasms. Such mechanisms have been thought to also interfere with and affect endothelial function. Another provoking cause has been described as Kounis syndrome, with a similar pathogenesis to an inflammatory process. It is an allergic acute coronary syndrome that is mediated by mast cells and platelet hypersensitivity, leading to coronary artery vasospasm or atheromatous plaque erosion with rupture. Half of the reported cases have been linked to antibiotic exposure, thought to be due to endothelial nitric oxide synthase's direct toxic effects on endothelium. Our patient did not present with any of the above-listed risk factors associated with coronary vasospasm.

## Conclusions

Immunosuppression therapy influences many cellular adaptations, with the potential to increase mortality. Tacrolimus and other calcineurin inhibitors have been discussed in much literature to have harmful side effect profiles. We aim for this case report to highlight the potential of tacrolimus to induce acute coronary syndrome, with a provoking incidence of coronary vasospasm.
